# THE IMPACT OF DEMENTIA STIGMA IN RECRUITING PARTICIPANTS INTO RESEARCH STUDIES

**DOI:** 10.1093/geroni/igz038.2200

**Published:** 2019-11-08

**Authors:** Karen Rose, Ruth Palan Lopez

**Affiliations:** 1 University of Tennessee, Knoxville, Tennessee, United States; 2 University of Tennessee, Knoxville, Knoxville, Tennessee, United States

## Abstract

Despite efforts to increase awareness and education about Alzheimer’s disease and other dementias, persons with dementia and their family caregivers experience stigma. Dementia related stigma is associated with negative repercussions for those with the disease and their family caregivers. In our prior work, we identified shame as a mechanism by which stigma is enacted and results in isolating and delaying access to supportive services for family caregivers. As such, stigma may influence decisions to participate in research studies. Healthcare providers, friends and family, and society, in general, play roles that further perpetuate stigma in dementia. Best practices from the literature, coupled with our experiences and findings in recruiting persons with dementia and their family caregivers in to research studies will be examined.

